# Novel Approaches to Lipid Management: Beyond Statins and PCSK9 Inhibitors

**DOI:** 10.14740/jocmr6523

**Published:** 2026-03-26

**Authors:** Jay Patel, Samarth Shah, Alayka Reddy, Deep Prajapati, Abhijit Pandya, Sumit Sawhney

**Affiliations:** a Department of Biomedical Engineering, Florida Atlantic University, Boca Raton, FL, USA; b Department of Technology and Clinical Trials, Advanced Research LLC, Pompano Beach, FL, USA; c Oncology and Hematology Associates of West Broward, Coral Springs, FL, USA

**Keywords:** Cholesterol, Lipid, Dyslipidemia, Statin, PCSK9 inhibitor, Cardiovascular, Lipid metabolism, Gene-based lipid therapy, Low-density lipoprotein

## Abstract

The statins remain the foundation of lipid management because they lower low-density lipoprotein cholesterol (LDL-C) and prevent cardiovascular events, and guidelines recommend stepwise intensification, often with ezetimibe first, when targets are not met or when intolerance limits dosing. This review introduces a mechanism-first, phenotype-guided framework that links add-on therapies to the dominant driver of residual risk, LDL-C, triglyceride-rich lipoproteins, elevated lipoprotein(a), or inherited dyslipidemia while integrating trial evidence with clinical practicality. Proprotein convertase subtilisin/kexin type 9 (PCSK9) monoclonal antibodies remain the best-validated add-on for very high-risk patients. FOURIER and ODYSSEY OUTCOMES demonstrated event reduction with evolocumab or alirocumab on background statin therapy. For patients who cannot tolerate adequate statin doses, bempedoic acid provides liver-selective inhibition of adenosine triphosphate (ATP)-citrate lyase, and CLEAR Outcomes showed fewer major cardiovascular events in statin-intolerant populations. Inclisiran extends PCSK9 pathway suppression through hepatic small interfering RNA (siRNA) and enables durable LDL-C reduction with twice-yearly maintenance dosing, offering an adherence-oriented alternative while outcomes data mature. Angiopoietin-like protein 3 (ANGPTL3)-directed therapies (evinacumab and investigational RNAi agents such as zodasiran) lower atherogenic lipoproteins through largely LDL receptor independent biology. They expand options for refractory disease, including homozygous familial hypercholesterolemia. Apolipoprotein C-III (ApoC-III) inhibitors (olezarsen and plozasiran) drive large triglyceride reductions that can be decisive in severe hypertriglyceridemia and pancreatitis-prone syndromes. Next-generation cholesteryl ester transfer protein (CETP) inhibition (notably obicetrapib) has re-emerged as an oral strategy with substantial lipid effects as outcomes programs progress. High-dose eicosapentaenoic acid (EPA) (icosapent ethyl) has the clearest triglyceride-focused outcomes signal; REDUCE-IT showed significant ischemic event reduction in statin-treated patients with persistent hypertriglyceridemia. Early *in vivo* PCSK9 gene-editing is considered a potential one-time approach, though safety and durability concerns remain unresolved. Implementation remains rate-limiting. Costs, prior authorization, variable coverage, distribution, injection logistics, and adherence barriers delay initiation and erode persistence. Uninsured patients may face prohibitive out-of-pocket expenses without assistance pathways.

## Introduction

### Lipids: definitions and core biological roles

Lipids comprise a broad category of hydrophobic or amphipathic compounds such as fats, oils, waxes, and certain fat-soluble vitamins that do not dissolve in water but readily mix with organic solvents [1]. They are vital to numerous physiological functions, including long-term energy storage, maintenance of cellular architecture, and facilitation of signal transduction [1]. As integral elements of biological membranes, lipids contribute to membrane integrity and regulate fluidity, ensuring proper cellular function [1]. Additionally, they serve as key precursors in the synthesis of hormones and play essential roles in intracellular communication. Their unique amphipathic properties enable the formation of lipid bilayers, which are critical for maintaining cellular compartmentalization and supporting organelle function [1].

### Lipid classification framework

Lipids are systematically classified based on their molecular structure and physiological roles [2]. The LIPID MAPS consortium outlines eight primary categories: fatty acyls, glycerolipids, glycerophospholipids, sphingolipids, sterol lipids, prenol lipids, saccharolipids, and polyketides [2]. Each of these groups comprises distinct lipid types with specialized biological functions. For example, glycerophospholipids are fundamental to the architecture of cellular membranes, whereas sterol lipids, such as cholesterol, not only contribute to membrane integrity but also act as biochemical precursors to steroid hormones [2].

### Overview of lipid metabolism and key biomarkers

Lipid metabolism encompasses complex pathways responsible for the synthesis, transport, and breakdown of lipids within the body [3]. A key catabolic process, lipolysis, involves the hydrolysis of triglycerides (TGs) into glycerol and free fatty acids, providing a major source of energy, particularly during fasting [3]. This process is tightly regulated by enzymes such as adipose triglyceride lipase, hormone-sensitive lipase (HSL), and monoglyceride lipase [3]. At the molecular level, transcription factors such as sterol regulatory element-binding proteins (SREBPs) regulate the expression of genes critical to lipid biosynthesis and homeostasis. Recent advances have identified several biomarkers, including proprotein convertase subtilisin/kexin type 9 (PCSK9), ApoC-III, and small dense low-density lipoprotein (sd-LDL), which have emerged as key indicators of dyslipidemia and cardiovascular risk [3]. These markers underscore the multifaceted nature of lipid regulation and its relevance in the pathogenesis of metabolic and cardiovascular diseases (CVD) [3].

### Lipid profile testing and clinical interpretation

A lipid profile is a routine blood test used to determine an individual’s lipid levels and quantify his or her risk for CVD [4]. The routine procedure consists of an examination for total cholesterol, low-density lipoprotein cholesterol (LDL-C), high-density lipoprotein cholesterol (HDL-C), very low-density lipoprotein (VLDL) cholesterol, and TGs. LDL-C, commonly labelled as “bad” cholesterol, is linked to the formation of arterial plaque and the development of atherosclerosis [4]. In contrast, HDL-C, often referred to as “good” cholesterol, helps remove LDL-C from the bloodstream [4]. Elevated TGs are also a problem, as they are associated with arterial stiffness and an increased rate of cardiovascular events. Regular monitoring of these lipid parameters is needed to early identify and control lipid-associated cardiovascular risk [4].

### Why novel lipid-lowering therapies are needed

**Figure 1:**
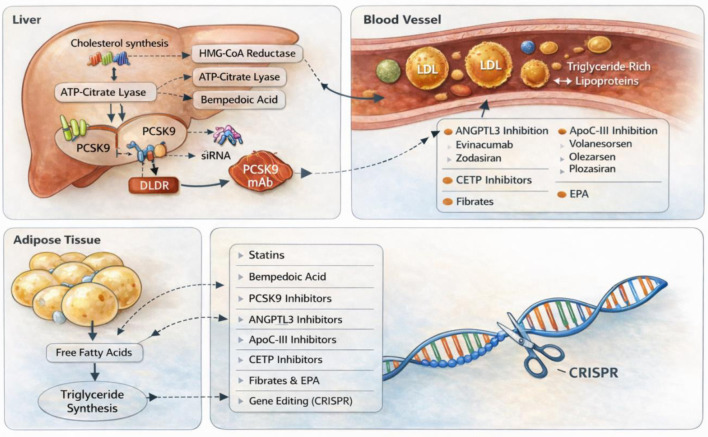
Mechanism-based overview of lipid metabolism and therapeutic targets. ATP: adenosine triphosphate; ANGPTL3: angiopoietin-like protein 3; ApoC-III: apolipoprotein C-III; CETP: cholesteryl ester transfer protein; CRISPR: clustered regularly interspaced short palindromic repeats; EPA: eicosapentaenoic acid; HMG-CoA: 3-hydroxy-3-methylglutaryl coenzyme A; LDL: low-density lipoprotein; LDLR: low-density lipoprotein receptor; PCSK9: proprotein convertase subtilisin/kexin type 9; siRNA: small interfering ribonucleic acid; TGs: triglycerides.

Effective control of lipid levels is fundamental to preventing atherosclerotic cardiovascular disease (ASCVD) [5]. Statins have been the first-line therapy for decades, dramatically reducing LDL-C and cardiovascular events [5]. More recently, monoclonal antibody PCSK9 inhibitors revolutionized care for very high-risk and familial hypercholesterolemia (FH) patients by enabling even greater LDL-C reductions [5]. From August 2021 through August 2023, data from the US National Center for Health Statistics indicate that 11.3% of adults aged 20 years and older had elevated total cholesterol levels (≥ 240 mg/dL) [6]. The highest prevalence was observed in the 40–59 age group (16.7%), followed by adults aged 60 and above (11.3%) and those aged 20–39 (6.0%) [6]. Furthermore, 13.8% of adults exhibited low levels of HDL-C defined as < 40 mg/dL, with men being disproportionately affected (21.5%) compared to women (6.6%) [6]. Notably, the occurrence of low HDL-C declined progressively with age across all demographic groups [6]. These statistics emphasize the continued importance of targeted lipid management interventions to reduce CVD risk [6]. Even though statins and monoclonal-antibody PCSK9 inhibitors are commonly prescribed, many patients, particularly those with FH, statin intolerance, or high cardiovascular risk, do not meet LDL-C targets and still have a substantial residual risk [7]. The figure summarizes in short regarding lipid metabolism across the liver, blood vessels, and adipose compartments, alongside current and emerging therapeutic approaches for dyslipidemia (Fig. 1).

Numerous mechanism-based treatments targeting different facets of lipid metabolism have been developed as a result of the current unmet need. These consist of angiopoietin-like protein 3 (ANGPTL3) inhibition (evinacumab, zodasiran), microsomal triglyceride transfer protein inhibition (lomitapide), adenosine triphosphate (ATP)-citrate lyase inhibition (bempedoic acid), small interfering RNA (siRNA) therapy (inclisiran), antisense oligonucleotide (ASO) (pelacarsan), cholesteryl ester transfer protein (CETP) inhibitors, apolipoprotein C-III (ApoC-III) inhibitors, and gene therapy [7]. For patients with persistent hypercholesterolemia or statin-associated side effects, these medications offer tailored approaches; nevertheless, their use requires careful consideration of cost, safety, and long-term outcome data [7]. In the past few years, pivotal clinical trials and regulatory approvals have brought these novel therapies to the forefront. This article highlights ongoing clinical trials, examines the mechanisms, efficacy, and safety profiles of new medications, and explores how they might enhance current treatments for individualized lipid management beyond conventional statins and PCSK9 inhibitors.

## Methods

### Literature search strategy

A narrative literature review search was conducted using PubMed and Google Scholar to identify relevant studies for this review. The search covered publications from 2000 to June 2025. Search strings included combinations of terms such as “lipid management,” “novel lipid-lowering therapy,” “bempedoic acid,” “inclisiran,” “angiopoietin like protein 3 (ANGPTL3),” “apolipoprotein C-III (APOC3),” “cholesteryl ester transfer protein (CETP) inhibitors,” “eicosapentaenoic acid (EPA),” and “lipoprotein(a).” Additional references were identified by reviewing the bibliographies of included articles.

### Study selection

Studies were selected for relevance to clinical lipid management, FH, hypertriglyceridemia, lipoprotein(a) (Lp(a)), and mechanism-based precision therapy. Foundational studies on lipid biology, screening, and statin pharmacology were also included to provide clinical context.

### Data extraction

Data extraction was performed independently by four authors using a standardized approach. Extracted variables included study design, population characteristics, interventions, comparators, follow-up duration, lipid effects, cardiovascular outcomes, safety findings, and key points, such as relative risk (RR) reductions, confidence intervals (CIs), and P values. Any discrepancies in study selection or extracted information were resolved through discussion among the authors until consensus was reached.

## Dyslipidemia: Epidemiology, Risk Factors, Screening, and Clinical Impact

### Definition and determinants of dyslipidemia

Dyslipidemia is a significant and modifiable risk factor for CVDs, characterized by abnormal levels of blood lipids and lipoproteins [8]. Immutable factors such as increasing age and inherited susceptibility, for example, a family history of FH or other monogenic lipid disorders, raise an individual’s baseline risk of dyslipidemia [8]. However, most of the population burden is driven by changeable determinants: dietary patterns high in saturated fats and simple sugars, low levels of physical activity, and excess adiposity (particularly central obesity) all promote an atherogenic lipid profile, while heavy alcohol use and tobacco smoking further worsen lipid and cardiovascular risk [8]. Several common medical conditions (notably insulin resistance and type 2 diabetes, hypothyroidism, and cortisol excess from Cushing syndrome) and organ dysfunction (nephrotic syndrome, chronic kidney disease, cholestatic liver disease) can cause or amplify lipid abnormalities but are often at least partly treatable [8].

Certain medications, for example, systemic corticosteroids, some retinoids, particular diuretics and hormonal agents, may adversely affect lipids and can be reviewed or modified when feasible [8]. Finally, socioeconomic and behavioral factors (lower income or education, adverse working or living conditions) shape exposure to many of these risks at the population level and are important targets for prevention efforts [8].

### Screening recommendations and testing intervals

The USPSTF’s 2023 recommendation states that existing evidence is inadequate to support routine universal lipid screening in asymptomatic children and adolescents aged 20 years or younger; consequently, it does not endorse a specific starting age or a fixed testing interval for the general pediatric population [9]. The multisociety Guideline on Management of Blood Cholesterol recommends targeted testing in children with a familial history of premature CVD or documented dyslipidemia as early as age 2, and advocates for universal lipid screening at ages 9–11 years and again at 17–21 years [9].

The USPSTF favors targeted, age-based screening over universal testing: it strongly recommends lipid screening for men ≥ 35 and for women ≥ 45 who have elevated coronary heart disease (CHD) risk, and it advises screening men 20–35 and women 20–45 when cardiovascular risk factors are present; in low-risk younger adults, the Task Force makes no routine recommendation. Assessment should begin with a standard lipid panel, since routine TG screening was not endorsed [9]. For low-risk individuals, repeating tests about every 5 years is reasonable, with shorter or longer intervals determined by results and changing risk [9]. Individuals are considered at elevated risk for CHD when major cardiovascular risk factors are present, including elevated LDL-C, hypertension, diabetes mellitus, cigarette smoking, obesity, and a family history of premature CVD. These factors significantly increase the likelihood of atherosclerotic cardiovascular events and warrant early clinical evaluation. Therefore, lipid screening is recommended in adults with these risk factors and in individuals with a family history suggestive of inherited lipid disorders [3, 5]. Lipid profiles should be reassessed 4–12 weeks after initiation or modification of lipid-lowering therapy and subsequently every 3–12 months to monitor therapeutic response and adherence [8].

### Clinical consequences

Dyslipidemia drives ASCVD and related events, most notably myocardial infarction (MI), unstable coronary syndromes, and ischemic stroke [10]. An elevated lipid burden also raises the likelihood of recurrent coronary events and long-term cardiovascular mortality, and it contributes to peripheral arterial disease and other manifestations of systemic atherosclerosis [10]. Extremely high TG concentrations (typically > 500 mg/dL) introduce a separate, acute risk by markedly increasing the chance of pancreatitis, so such elevations require urgent management to reduce that risk [10].

### Treatment foundation: lifestyle and risk-based pharmacotherapy

All patients should initiate or enhance lifestyle modifications, adopt a heart-healthy diet (like Mediterranean), engage in regular physical activity, achieve weight reduction if necessary, and cease smoking [10]. Drug therapy is indicated when the anticipated benefit outweighs harms, with the strongest priority given to secondary prevention. Patients with established ASCVD should start a high-intensity statin unless there are clear contraindications [10]. Individuals with very high baseline LDL-C (generally > 190 mg/dL, often reflecting heterozygous FH) warrant prompt initiation of statin treatment and consideration of more intensive LDL-lowering measures. For adults with diabetes aged roughly 40–75 years whose LDL-C falls between about 70 and 189 mg/dL, at least a moderate-intensity statin is recommended, with the intensity tailored to the person’s overall risk profile [10]. In other primary prevention scenarios (LDL-C, 70–189 mg/dL without diabetes or known ASCVD), treatment decisions should be guided by an estimated 10-year ASCVD risk (using validated calculators); statin therapy is generally offered when the calculated absolute risk crosses the threshold at which expected benefit exceeds potential harms, and initiation should follow shared decision making with the patient [10]. Commence by advocating for and facilitating healthy lifestyle modifications, subsequently assess the patient’s atherosclerotic cardiovascular risk, and when appropriate, initiate pharmacological intervention through shared decision making to discuss anticipated benefits, potential adverse effects, and the financial ramifications of lipid-lowering medications.

In addition to lipid thresholds and clinical risk factors, contemporary guidelines emphasize structured cardiovascular risk assessment to refine treatment decisions in primary prevention. Several validated risk prediction models are used internationally to estimate the probability of future cardiovascular events. In the United States, the Pooled Cohort Equations (PCE) are widely recommended for estimating the 10-year risk of ASCVD, incorporating variables such as age, sex, race, total cholesterol, HDL-C, systolic blood pressure, diabetes status, smoking history, and antihypertensive therapy. These equations allow clinicians to categorize patients into risk strata (low, borderline, intermediate, and high risk) and determine when statin therapy or additional preventive interventions may provide a net clinical benefit.

Other widely recognized risk models include the Framingham Risk Score (FRS) and the Systematic Coronary Risk Evaluation (SCORE) system, both of which estimate cardiovascular risk using similar demographic and metabolic parameters. Although these tools share common predictors, they differ in the outcomes they predict and the populations for which they were developed [5, 8]. The FRS estimates the risk of CHD events, whereas the PCE predict broader ASCVD outcomes, including MI and stroke [8].

The SCORE system, commonly used in European guidelines, focuses on fatal cardiovascular events and incorporates regional calibration to account for geographic variations in risk. Integrating these models with clinical judgment enables a more individualized approach to therapy, particularly in borderline or intermediate-risk individuals where additional “risk-enhancing factors” (such as family history of premature CVD, metabolic syndrome, chronic kidney disease, inflammatory disorders, or persistently elevated LDL-C) may justify earlier pharmacologic intervention. This risk-based framework strengthens preventive strategies by aligning treatment intensity with a patient’s overall cardiovascular risk profile and supporting shared decision-making between clinicians and patients [5, 8].

## Established Pharmacotherapies

### Statins

#### Mechanism of action

Statins function by competitively inhibiting hepatic 3-hydroxy-3-methylglutaryl-coenzyme A (HMG-CoA) reductase, thereby reducing intrahepatic cholesterol synthesis and enhancing LDL receptor expression to facilitate the clearance of circulating LDL-C [11].

#### Agents and pharmacokinetics

The most commonly prescribed agents include atorvastatin, simvastatin, rosuvastatin, pravastatin, fluvastatin, lovastatin, and pitavastatin; they differ in lipophilicity and pharmacokinetics, which can affect drug-drug interactions and tolerability [12]. Metabolism is predominantly hepatic: simvastatin, lovastatin, and atorvastatin are extensively metabolized by CYP3A4, fluvastatin by CYP2C9, whereas rosuvastatin and pravastatin exhibit reduced CYP-mediated metabolism and increased renal or biliary excretion (rosuvastatin demonstrates a significant renal elimination fraction, while atorvastatin’s renal clearance is minimal) [12].

#### Efficacy and comparative data

Numerous meta-analyses of randomized trials demonstrate the therapeutic significance of LDL reduction with statin therapy: for every 1.0 mmol/L (39 mg/dL) decrease in LDL-C, major vascular events decrease by approximately 20–21% on average across various groups [13]. Jaam et al performed a systematic review and meta-analysis searching the literature through December 2021 and identified 44 head-to-head randomized trials and cohort studies that directly compared high-intensity statin regimens across varied adult patient populations [14]. The pooled evidence showed that high-intensity statins reliably lower LDL-C by ≥ 50% from baseline, with similar overall safety profiles across agents (adverse reactions rose with higher doses) [14]. In the quantitative comparison of atorvastatin 80 mg versus rosuvastatin 40 mg, rosuvastatin produced a significantly greater LDL reduction, leading the authors to conclude that while all high-intensity statins are effective, rosuvastatin appears modestly superior to atorvastatin for LDL lowering [14].

#### Safety and limitations

Statins possess certain limitations; a modest yet consistent increased risk of new onset diabetes has been documented (approximately 9–12% relative increase in some meta-analysis), and muscle-related issues, from myalgia to infrequent myopathy/rhabdomyolysis, affect a minority of patients [15]. Consequently, drug-drug interactions (particularly through CYP3A4) and variable tolerability impact the selection and dosing of these agents [15]. Statins have also been linked to a modest increase in the risk of incident diabetes, particularly among individuals with preexisting metabolic risk factors, though the overall cardiovascular benefits generally outweigh this risk [15]. Additionally, concerns regarding liver enzyme elevations or potential effects on hepatic steatosis have been evaluated, with evidence suggesting that statins are generally safe and may even confer benefit in certain liver conditions [16]. Overall, while side effects can occur in a minority of patients, the prevalence of serious adverse events remains low, and statins continue to provide substantial net benefit in the prevention of CVD.

#### Statins in CVD and non-alcoholic fatty liver disease (NAFLD)/non-alcoholic steatohepatitis (NASH)

Statins play a central role in both the prevention and long-term management of CVD by targeting the underlying pathophysiology of atherosclerosis. By inhibiting hepatic cholesterol synthesis and enhancing clearance of circulating LDL-C, statins reduce lipid accumulation within arterial walls and slow the progression of atherosclerotic plaque formation. Beyond lipid-lowering, these agents also exert additional vascular benefits, including stabilization of existing plaques, improvement of endothelial function, and attenuation of inflammatory processes that contribute to plaque rupture and thrombosis. Through these combined mechanisms, statins have consistently demonstrated substantial reductions in major adverse cardiovascular events (MACEs) such as MI, ischemic stroke, and cardiovascular mortality [11].

Consequently, they remain crucial for both primary prevention in individuals with elevated cardiovascular risk and secondary prevention in patients with established atherosclerotic disease, forming the foundation upon which other lipid-lowering therapies are often added to achieve optimal cardiovascular risk reduction [11, 13]. That is the reason statins are regarded as the primary pharmacologic therapy for individuals with dyslipidemia or an increased risk of CVD due to their well-established efficacy in lowering LDL-Cand reducing cardiovascular events [8, 10]. Moreover, the widespread availability of generic statin formulations has improved accessibility and affordability, making these medications among the most cost-effective options for lipid-lowering treatment in everyday clinical practice [10]. Fatima et al performed a systematic review and meta-analysis of 14 studies comprising about 1,247,503 participants to evaluate the effect of statin therapy on NAFLD and NASH [16]. Statin exposure was associated with a lower likelihood of developing NAFLD (odds ratio (OR) 0.69; 95% CI, 0.57–0.84; P = 0.0002) and produced significant improvements in liver enzymes, alanine aminotransferase (ALT) (weighted mean difference (WMD) −27.28 U/L; 95% CI, −43.06 to −11.51; P = 0.0007), aspartate aminotransferase (AST) (WMD −10.99 U/L; 95% CI, −18.17 to −3.81; P = 0.003), and gamma-glutamyl transferase (GGT) (WMD −23.40 U/L; 95% CI, −31.82 to −14.98; P < 0.00001). Histologic measures also favored statin users, with reductions in steatosis (standardized mean difference (SMD) −2.59; 95% CI, −4.61 to −0.56; P = 0.01), NAFLD activity score (WMD −1.03; 95% CI, −1.33 to −0.74; P < 0.00001), and necroinflammation (WMD −0.19; 95% CI, −0.26 to −0.13; P < 0.00001) [16]. The pooled analysis suggested lower odds of significant fibrosis (OR 0.20; 95% CI, 0.04–0.95; P = 0.04), although the overall change in fibrosis stage was not statistically significant (WMD 0.07; 95% CI, −0.05 to 0.20; P = 0.27) [16]. The statins are associated with favorable biochemical and many histologic outcomes in NAFLD and with reduced risk of NAFLD development, but they call for larger prospective trials to confirm effects on fibrosis and long-term clinical endpoints [16].

### PCSK9-directed therapies

#### Mechanism of action

PCSK9-directed therapies effectively lower LDL-C by maintaining the function of hepatocyte LDL receptors [17]. Monoclonal antibodies, such as evolocumab and alirocumab, interact with circulating PCSK9 to inhibit receptor degradation [17].

#### Efficacy and comparative data

Sabatine et al concluded that, in patients with established ASCVD, add-on therapy with evolocumab produced a large mean LDL-C reduction (59% versus placebo) and led to a significant lowering of the trial’s primary composite cardiovascular outcome (hazard ratio (HR) 0.85; 95% CI, 0.79–0.92; P < 0.001) [17].

Schwartz et al concluded that alirocumab, when given after acute coronary syndrome, achieved similarly substantial LDL-C lowering (54–55%) and significantly reduced MACEs (HR 0.85; 95% CI, 0.78–0.93; P < 0.001), with the largest absolute risk reductions observed in participants with higher baseline LDL-C [18]. Real-world evidence indicated a prevalence of prior authorization denials and significant rates of prescription abandonment due to cost-sharing requirements; numerous plans mandated extensive documentation and reauthorization processes [19]. Injectable biologics administered subcutaneously every 2–4 weeks may lead to lower patient acceptance and adherence than oral medications [19]. Injection-site reactions and the requirement for continuous clinic or pharmacy processes can serve as deterrents [19].

Evolocumab has demonstrated robust cardiovascular benefit in the FOURIER trial, a large randomized study involving patients with established ASCVD receiving background statin therapy. In this trial, evolocumab produced a 59% reduction in LDL-C from baseline compared with placebo, lowering the median LDL-C level from 92 to 30 mg/dL [17]. This substantial lipid reduction translated into a significant decrease in the primary composite cardiovascular endpoint, which included cardiovascular death, MI, stroke, hospitalization for unstable angina, or coronary revascularization (HR 0.85; 95% confidence interval (CI), 0.79–0.92; P < 0.001) [17]. The key secondary endpoint, consisting of cardiovascular death, MI, or stroke, was also significantly reduced (HR 0.80; 95% CI, 0.73–0.88; P < 0.001) [17]. These findings established evolocumab as an effective adjunct to statin therapy for high-risk patients requiring further LDL-C lowering beyond conventional treatment targets.

Similarly, the ODYSSEY OUTCOMES trial confirmed the clinical value of alirocumab in patients with recent acute coronary syndrome who remained at elevated risk despite intensive or maximally tolerated statin therapy. Alirocumab achieved an approximate 54–55% reduction in LDL-C, with on-treatment LDL-C levels substantially lower than those observed in the placebo group [18]. Over a median follow-up of 2.8 years, alirocumab significantly reduced the risk of MACEs, including CHD death, nonfatal MI, fatal or nonfatal ischemic stroke, or unstable angina requiring hospitalization (HR 0.85; 95% CI, 0.78–0.93; P < 0.001) [18]. Notably, the absolute benefit was greatest among patients with higher baseline LDL-C levels, underscoring the importance of intensified LDL-C lowering in individuals at particularly high residual risk after acute coronary events [18]. Together, FOURIER and ODYSSEY OUTCOMES provide strong outcome-based evidence that PCSK9 inhibition not only produces profound LDL-C reduction but also meaningfully lowers cardiovascular event rates in secondary prevention populations.

### Statins and PCSK9 inhibitors in FH

The study by Versmissen et al demonstrated that statin therapy significantly reduces the risk of CHD in patients with FH, thereby endorsing statins as the first-line treatment for this demographic (HR 0.24; 95% CI, 0.18–0.30; P < 0.001) [20]. High-intensity statins are generally the first-line treatment for heterozygous FH, achieving average reductions in LDL-C of approximately 40–50% and often serving as the basis for further therapeutic escalation when lipid targets are not met [21]. Patients who do not achieve target LDL-C levels despite maximally tolerated statin therapy (with or without ezetimibe) have shown significant LDL-C reductions with PCSK9 monoclonal antibodies in randomized trials [22]. In RUTHERFORD-2, evolocumab resulted in a mean LDL-C reduction of approximately 59% compared with placebo (both dosing regimens; P < 0.0001) [22]. Similarly, in ODYSSEY HIGH FH, alirocumab demonstrated a change of −45.7% versus −6.6% with placebo (difference −39.1%; P < 0.0001), markedly increasing the percentage of patients achieving predefined LDL targets [23].

In homozygous FH, response variability is greater and is influenced by the remaining activity of LDL receptors; TESLA Part B indicated a significant yet smaller average reduction in LDL-C at 12 weeks (30.9% compared to placebo; 95% CI, −43.9 to −18.0; P < 0.0001) [24]. In summary, statins remain the primary treatment for genetic hypercholesterolemia, whereas PCSK9 inhibitors offer significant additional LDL-lowering effects that enhance goal achievement in heterozygous FH and may be advantageous for certain homozygous patients based on genotype and receptor activity [20]. Although statins and PCSK9 inhibitors form the therapeutic foundation for LDL lowering and have demonstrable outcome benefits, limitations in tolerability, incomplete LDL control in some genetic disorders, cost and convenience concerns, and residual cardiovascular risk have driven the development of several new agents that target distinct pathways in lipoprotein metabolism.

## Novel and Emerging Lipid-Lowering Agents

### Bempedoic acid

Bempedoic acid is an oral medication that reduces cholesterol synthesis through inhibition of ATP-citrate lyase, an enzyme that acts upstream of HMG co-enzyme A (CoA) reductase [25]. In contrast to statins, this compound functions as a prodrug, primarily activated in the liver rather than in muscle, thereby reducing the incidence of muscle-related side effects [25].

Lincoff et al performed a comparative analysis applying the Cholesterol Treatment Trialists (CTTC) methodology to outcomes from the CLEAR Outcomes randomized trial (13,970 patients, statin-intolerant or at high cardiovascular risk) to determine whether the cardiovascular benefit per unit LDL-C lowering with bempedoic acid mirrors that seen with statins [26]. Over a median follow-up, the incidence of a first major vascular event was lower in the bempedoic acid arm (703 events, 10.1%) than in placebo (816 events, 11.7%), yielding an unadjusted HR of 0.85 (95% CI, 0.77–0.94); when normalized to a 1.0 mmol/L reduction in LDL-C, the treatment effect was HR 0.75 (95% CI, 0.63–0.90), which the authors found to be comparable to the CTTC-reported statin rate ratio of 0.78 for the same standardized LDL-C decrement [26]. These results support the conclusion that the cardiovascular risk reduction achieved with bempedoic acid is similar, on a per-unit LDL-lowering basis, to that observed with statins [26].

The authors report a prespecified total events analysis of the CLEAR Outcomes randomized trial, which enrolled 13,970 adults at high cardiovascular risk who were either unable or unwilling to take guideline recommended statin doses. In follow-up studies, bempedoic acid demonstrated a significant reduction in the overall incidence of major cardiovascular events. The MACE-4 composite endpoint, which includes cardiovascular death, nonfatal MI, nonfatal stroke, or coronary revascularization, occurred less frequently with bempedoic acid (HR 0.80; 95% CI, 0.72–0.89; P < 0.001). Additionally, the more specific MACE-3 endpoint, comprising cardiovascular death, nonfatal MI, and nonfatal stroke, also showed a reduction (HR 0.83; 95% CI, 0.73–0.93; P = 0.002). The drug demonstrated substantial reductions in MI (HR 0.69; 95% CI, 0.58–0.83; P < 0.001) and coronary revascularization (HR 0.78; 95% CI, 0.68–0.89; P < 0.001); however, no statistically significant effect was observed on stroke (HR 0.80; 95% CI, 0.63–1.03). The authors conclude that, in a statin intolerant high-risk population, bempedoic acid significantly reduced the total burden of cardiovascular events by lowering LDL-C [27].

The study presents a prespecified CLEAR Outcomes subset comprising 6,177 participants with obesity, with a median follow-up of 40.7 months. At 6 months, bempedoic acid reduces LDL-C by 22.5% and high-sensitivity C-reactive protein (hs-CRP) by 23.2% [28]. Treatment resulted in a 23% reduction in the composite MACE-4 endpoint (HR 0.77; 95% CI, 0.67–0.89), with significant decreases observed in MI (HR 0.68; 95% CI, 0.53–0.86), coronary revascularization (HR 0.76; 95% CI, 0.63–0.92), and stroke (HR 0.64; 95% CI, 0.45–0.89) [28]. The authors conclude that, among people with obesity, bempedoic acid lowered LDL-C and inflammation and significantly reduced major cardiovascular events [28].

The adverse effects most consistently associated with bempedoic acid include an elevated risk of gout, a higher incidence of cholelithiasis, and slight increases in creatinine and hepatic enzyme levels [27–29]. These observations are derived from randomized trials, pooled analyses, and the extensive CLEAR Outcomes program [27–29].

### Inclisiran

Inclisiran is a GalNAc-conjugated siRNA that selectively targets hepatic PCSK9 messenger RNA [30]. After subcutaneous (SC) administration, the GalNAc ligand mediates uptake into hepatocytes via the asialoglycoprotein receptor; the siRNA is loaded into the RNA-induced silencing complex (RISC), which cleaves PCSK9 mRNA and thereby suppresses hepatic PCSK9 synthesis [30]. Reduced circulating PCSK9 permits greater LDL receptor recycling at the hepatocyte surface, increasing hepatic LDL-C uptake and producing sustained reductions in plasma LDL-C [30].

Taub et al conducted VICTORION-Mono, a multicenter, international, randomized, double blind phase-three trial that enrolled adults without ASCVD, diabetes, or FH to evaluate inclisiran monotherapy over a 6-month treatment period (150 days) with a 30-day safety follow-up [30]. The study randomized 350 participants (2:1:1) to inclisiran (n = 174), ezetimibe (n = 89) or placebo (n = 87); mean age was 46.1 years, 62.6% were female, baseline LDL-C averaged 135.4 mg/dL, mean body mass index (BMI) was 29.8 kg/m^2^, and median 10-year ASCVD risk was 2.2% [30]. Inclisiran produced a mean LDL-C reduction of −46.5% at day 150 versus +1.4% with placebo and −11.2% with ezetimibe; the between-group differences were large and highly significant (inclisiran vs. placebo −47.9%, and vs. ezetimibe −35.4%; P < 0.0001 for both comparisons) [30]. Other lipid parameters, including PCSK9 and Lp(a), improved favorably with inclisiran, and the agent was well tolerated with no new safety signals observed during the 6-month period [30]. In summary, the authors conclude that inclisiran as monotherapy delivers robust and durable LDL-C lowering and a reassuring short-term safety profile compared with placebo and ezetimibe in low-risk adults not previously receiving lipid-lowering therapy [30].

Basit et al performed a systematic review and meta-analysis of randomized controlled trials (RCTs) by searching PubMed/MEDLINE, Embase, and the Cochrane Library through July 2024 to compare inclisiran versus control (placebo or standard therapy) [31]. The pooled analysis included eight RCTs with 5,016 participants (Individual trial locations and mean participant ages are reported within each trial; a single pooled mean age is not provided in the abstract) [31]. Inclisiran produced a large and statistically significant pooled reduction in LDL-C (mean difference (MD) −50.42%; 95% CI, −56.15 to −44.70; P < 0.05) and a marked reduction in circulating PCSK9 (MD −78.57%; 95% CI, −81.64 to −75.50). Total cholesterol and apolipoprotein B (apoB) were also significantly reduced (MDs −31.22% and −41.47%, respectively; 95% CIs reported in the paper) [31]. Major clinical safety endpoints all-cause mortality, cardiovascular death, MACEs, MI, stroke, and serious adverse events were similar between inclisiran and control (pooled P > 0.05). The authors conclude that inclisiran provides large, durable reductions in LDL-C, PCSK9, and apoB without an increased risk of serious adverse events in randomized trials [31].

The study performed a systematic review and meta-analysis conducted by a Brazilian team at the State University of Ponta Grossa that searched PubMed, Scopus, and Web of Science, pooling 32 trials (30,718 patients) and analyzing lipid endpoints at 12 and 24 weeks to compare inclisiran and alirocumab [32]. In pooled analyses, alirocumab 75 mg produced a larger LDL-C reduction (−51.54%, 95% CI, −53.43 to −49.66) than inclisiran 300 mg (−41.34%, 95% CI, −50.30 to −31.34), the between-group difference reaching nominal significance (P = 0.05); alirocumab (both 75 and 150 mg) was also superior to inclisiran for total cholesterol (P < 0.01) and for TGs in several dose comparisons (P = 0.02 and P = 0.04) [32]. There was no significant difference between agents for the reduction of lipoprotein-a (P = 0.26 and P = 0.16 for the reported comparisons). The authors conclude that, over 24 weeks, alirocumab yields greater improvement in key lipid parameters, especially LDL-C, total cholesterol, and TGs, than inclisiran [32].

Rai et al conducted a systematic review and meta-analysis of four ORION clinical trials to compare the efficacy of inclisiran in patients with heterozygous versus homozygous FH [33]. They found that inclisiran (300 mg subcutaneously) produced substantially larger percentage reductions in atherosclerotic lipid measures (LDL-C, total cholesterol, apoB, and non-HDL-C) in heterozygous FH patients than in homozygous FH patients (all differences statistically significant, P < 0.05) [33]. By contrast, reductions in circulating PCSK9 were large in both groups, and the between-group difference for PCSK9 did not reach statistical significance (P = 0.20). The authors conclude that inclisiran reduces atherogenic lipid parameters to a significantly greater extent in heterozygous FH than in homozygous FH [33].

### ANGPTL3 targeting

#### Evinacumab

Evinacumab is a fully human monoclonal antibody directed against ANGPTL3, a hepatic regulator that suppresses the activity of lipoprotein lipase (LPL) and endothelial lipase (EL) [34]. Inhibition of ANGPTL3 restores LPL and EL function, thereby enhancing the hydrolysis of VLDL and intermediate-density lipoproteins (IDL) and facilitating remnant clearance through non-LDL receptor pathways [34]. Some studies have demonstrated increased fractional catabolic rates of apoB-containing lipoproteins and, in some cases, reduced VLDL apoB production [34]. Collectively, these mechanisms decrease remnant-to-LDL conversion flux and account for the LDL receptor-independent reduction in LDL-C observed with evinacumab therapy [34].

Rangwala et al performed a systematic review and meta-analysis of RCTs with literature searched through 2023, pooling four RCTs for a total of 270 patients that compared evinacumab (various regimens, notably high dose 15 mg) versus placebo; the included trials reported primary endpoints at roughly week 16 to week 24 [34]. The meta-analysis did not provide a single pooled mean age (included trials enrolled adults, and the homozygous FH trial allowed patients ≥ 12 years), with trial-level mean ages generally in the mid-40s to mid-50s. In pooled results, evinacumab did not significantly reduce TGs (P = 0.16) but produced statistically significant reductions in total cholesterol (P = 0.02), HDL-C (P = 0.001), apoB (P = 0.03), and ApoC-III (P = 0.004), and a borderline/significant reduction in LDL-C (P = 0.05); pooled safety analysis showed no clear increase in adverse events versus placebo [34]. The authors conclude that high-dose evinacumab (15 mg) appears efficacious for lowering several atherogenic lipid markers with acceptable tolerability, while TG effects were not significant and further studies are needed to define long-term clinical outcomes and safety [34].

The author conducted a multicenter, double blind, randomized, placebo-controlled phase 2 trial that enrolled 272 adults with refractory hypercholesterolemia to evaluate SC and intravenous (IV) evinacumab; the primary (double blind) treatment period and primary endpoint were assessed through week 16 [35]. Patients (mean age 52.1 ± 12.9 years as reported in the literature) were randomized to several active regimens (SC evinacumab 450 mg weekly, SC 300 mg weekly, SC 300 mg every 2 weeks, IV 15 mg/kg every 4 weeks, IV 5 mg/kg every 4 weeks) or matching placebo while continuing background lipid-lowering therapy [35]. At week 16, the least squares mean differences in percent change from baseline in LDL-C versus placebo were: −56.0% (450 mg SC QW), −52.9% (300 mg SC QW), −38.5% (300 mg SC Q2W), −50.5% (IV 15 mg/kg Q4W) (all P < 0.001), and −24.2% (IV 5 mg/kg Q4W; P = 0.019), with serious adverse event rates varying across groups (3–16%) [35]. Overall, the trial showed that both SC and IV evinacumab produced rapid (by week 2) and substantial reductions in LDL-C versus placebo (largest reductions at the higher doses), supporting evinacumab as an effective LDL-lowering option for patients with refractory hypercholesterolemia on maximally tolerated background therapy; safety was acceptable in the 16-week period but with some serious adverse events reported [35].

During the 16-week double blind period, adverse events were common with evinacumab but generally low in severity for SC dosing; adverse effects occurred in 68%, 67%, and 82% of patients receiving 450 mg weekly, 300 mg weekly, and 300 mg every 2 weeks (vs. 54% with SC placebo), and for IV dosing, adverse effects occurred in 84% and 75% of patients receiving 15 and 5 mg/kg (vs. 70% with IV placebo) [35]. Events occurring in ≥ 5% of evinacumab recipients and more often than placebo included (SC) urinary tract infection (11% vs. 8%), injection site erythema (6% vs. 3%), arthralgia (5% vs. 3%), and myalgia (5% vs. 0%), and (IV) nasopharyngitis (12% vs. 6%), back pain (7% vs. 6%), limb pain (7% vs. 6%), dizziness (7% vs. 0%), fatigue (7% vs. 6%), nausea (7% vs. 0%), and abdominal pain (6% vs. 0%) [35]. Few high-grade events were observed, and there were no meaningful differences between SC and IV groups in rates of serious adverse events, treatment discontinuations, or prespecified adverse events of special interest [35]. Treatment-related discontinuations were rare (one SC case of dyspnea that resolved); there were two deaths in SC recipients with preexisting cardiac disease that investigators judged unrelated to drug; and one IV-treated patient experienced a treatment-related anaphylactic reaction (15 mg/kg) that resolved the same day after diphenhydramine; no deaths occurred in the IV groups [35]. In summary, ANGPTL3 inhibition is a novel strategy that can produce profound lipid lowering in scenarios previously deemed untreatable, exemplifying the move toward mechanism-specific therapies for extreme lipid disorders.

#### Zodasiran

Zodasiran is a GalNAc-conjugated RNA interference therapy that suppresses hepatic ANGPTL3 expression, thereby relieving inhibition of LPL and EL and targeting TG-rich lipoprotein metabolism [36].

In the phase two-b, double blind, dose-ranging ARCHES-two randomized trial, 204 adults with mixed hyperlipidemia (fasting TGs 150–499 mg/dL plus LDL-C ≥ 70 mg/dL or non-HDL-C ≥ 100 mg/dL) were assigned 3:1 to zodasiran 50, 100, or 200 mg subcutaneously on day 1 and week 12 or to placebo, with follow-up through week 36 and a primary endpoint of percent change in TGs at week 24 [36]. Zodasiran produced significant, dose-dependent placebo-adjusted TG reductions of 51%, 57%, and 63% at 50, 100, and 200 mg, respectively (all P < 0.001), along with decreases in non-HDL-C (−29%, −29%, −36%), apoB (−19%, −15%, −22%), and LDL-C (−16%, −14%, −20%) [36]. A transient rise in HbA1c was observed in participants with preexisting diabetes at the highest dose, while overall adverse event rates were similar to placebo in external summaries of the study and primarily reflected common intercurrent illnesses, with no platelet signal reported [36]. The authors concluded that ANGPTL3 silencing with zodasiran yields robust TG lowering and broader improvements in atherogenic lipoproteins at 24 weeks, supporting further outcomes-focused evaluation [36].

### TG-focused therapies

#### Fibrates

The author conducted a systematic review and meta-analysis of 12 RCTs that compared fibrates with a control group, involving a total of 53,231 participants (25,781 receiving fibrates; 27,450 in the control group) [37]. The trials included adults with dyslipidemia, frequently mixed dyslipidemia or diabetes, and documented both cardiovascular outcomes and lipid changes during treatment. Fibrates significantly reduced MACEs (RR 0.87, 95% CI, 0.81–0.94) [37]. Meta-regression analysis indicated that a 1 mmol/L reduction in LDL-C due to fibrates correlates with a decreased risk of MACE (RR 0.71, 95% CI, 0.49–0.94; P = 0.01) [37]. In contrast, alterations in TG levels did not demonstrate a significant association with MACE reduction (RR per 1 mmol/L TG reduction 0.96, 95% CI, 0.53–1.40; P = 0.86). The authors indicate that the cardiovascular benefits of fibrates in lipid management are likely mediated through LDL-C reduction, which accounts for the varied outcomes observed across different trials and populations [37].

Jakob et al compiled six randomized studies (n = 16,135) examining fibrates for primary prevention in people without established CVD (mean ages across trials 47.3–62.3 years; four trials exclusively enrolled patients with type 2 diabetes; mean follow-up duration was 4.8 years) [38]. The authors indicate that fibrates reduce TGs and alter atherogenic dyslipidemia; however, the review did not conduct a meta-analysis of lipid (TG/HDL/LDL) alterations, concentrating instead on clinical outcomes [38]. Pooled analyses indicated that fibrates diminished the composite outcome of CVD mortality, nonfatal MI, or nonfatal stroke (RR 0.84, 95% CI, 0.74–0.96), and decreased CHD mortality or nonfatal MI (RR 0.79, 95% CI, 0.68–0.92), while exhibiting no impact on overall mortality (RR 1.01, 95% CI, 0.81–1.26) or non-CVD mortality (RR 1.01, 95% CI, 0.76–1.35) [38]. Reporting of side effects was limited, with cessation due to adverse events used as a proxy, resulting in an imprecise estimate [38]. Additionally, trials examining fibrates in conjunction with statins (two studies) demonstrated no advantage in event outcomes. The study concludes that primary prevention with fibrates can modestly decrease cardiovascular and coronary events (absolute risk reductions < 1%) but does not affect mortality; the review emphasizes their function as TG-lowering agents while noting that event benefits were minimal and not apparent when used in conjunction with statins [38]. In pooled analyses, the authors report no excess risk of rhabdomyolysis with fibrates (RR 1.03) and no increase in gastrointestinal events (RR 0.90) compared with statin monotherapy; they also found no signal of greater harm when fibrates were combined with statins, though safety reporting varied across included trials [39]. In short, within the type 2 diabetes populations studied, fibrates did not show a higher rate of major adverse effects compared with statins, while providing only modest lipid changes [39].

The author synthesized 16 trials with 1,388 adults across mixed dyslipidemia settings (including diabetes, primary hypercholesterolemia, combined hyperlipidemia, primary hypertriglyceridemia, heart transplant recipients, and familial defective apoB) [40]. Intervention durations ranged from 8 to 24 weeks in parallel and cross-over designs, and drug regimens included fenofibrate, gemfibrozil, bezafibrate, and several statins at standard doses. The author performed a systematic review and meta-analysis of head-to-head randomized trials comparing fibrates and statins (and combination therapy) for their effect on Lp(a). The meta-analysis revealed that fibrates had a greater impact on lowering Lp(a) than statins (WMD −2.70 mg/dL, 95% CI, −4.56 to −0.84; P = 0.004) [40]. Additionally, the combination of fibrates and statins was found to have a greater effect on lowering Lp(a) than statin alone (WMD −1.60 mg/dL, 95% CI, −2.93 to −0.26; P = 0.019). The advantage of fibrates was even greater when baseline Lp(a) was 30 mg/dL or higher, and treatment durations of 12 weeks or more favored fibrates [40]. However, the main focus of the research is Lp(a) alterations; fibrates are lipid-lowering medications that lower TG-rich lipoproteins [40]. This mechanism may be related to Lp(a) changes; however, no pooled TG effect is mentioned in this report. In these RCTs, fibrates were found to be more effective than statins in lowering Lp(a), and adding a fibrate to a statin further enhanced Lp(a) reduction. The author noted that patients with elevated baseline Lp(a) levels saw the greatest benefits [40]. Fibrates reliably cut TGs and modestly reduce cardiovascular events, according to the studies we reviewed. However, the outcome advantages of fibrates appear more strongly associated with lowering LDL-C than TG.

#### ApoC-III inhibitors

ApoC-III is an inhibitor of LPL; high ApoC-III levels cause elevated TGs and are associated with pancreatitis and atherosclerosis in familial syndromes [41]. Inhibiting ApoC-III can dramatically reduce TG levels, which is especially beneficial for patients with hypertriglyceridemia at risk of pancreatitis and potentially for reducing residual cardiovascular risk related to TG-rich lipoproteins [41].

##### 1) Volanesorsen

Volanesorsen was the first antisense oligonucleotide targeting ApoC-III. Patients with familial chylomicronemia syndrome who received volanesorsen in the phase 3 APPROACH study showed significant reductions in TG levels (a 70–77% decrease from baseline, compared with an increase in the placebo group) [41]. Indeed, 77% of treated FCS patients experienced a reduction in TG levels to below 750 mg/dL, a threshold at which the risk of pancreatitis is significantly diminished [41]. Nonetheless, issues have occurred, necessitating monitoring due to significant side effects, including injection site reactions and thrombocytopenia [41].

##### 2) Olezarsen

This phase-2b, randomized, double blind, placebo-controlled trial included 154 adults from 24 North American sites, all of whom presented with either moderate hypertriglyceridemia (150–499 mg/dL) with heightened cardiovascular risk or severe hypertriglyceridemia (≥ 500 mg/dL) [42]. Participants on stable background lipid treatment were assigned to receive monthly SC olezarsen (50 or 80 mg) or placebo. At 6 months, the primary endpoint, the percentage change in TG levels, was met, with mean decreases of 49.3% and 53.1% in the 50 mg and 80 mg groups, respectively (P < 0.001 for both compared with placebo) [42]. Olezarsen also led to substantial decreases in ApoC-III, apoB, and non-HDL-C, although LDL-C remained unchanged [42]. In summary, monthly olezarsen resulted in significant decreases in TGs and atherogenic lipoproteins in patients with hypertriglyceridemia at elevated cardiovascular risk, accompanied by a favorable safety profile [42]. These findings underscore ApoC-III inhibition as a potentially effective treatment approach, while its impact on cardiovascular outcomes has yet to be established [42]. Clinically, ApoC-III inhibitors are niche but important: they are transforming care for rare diseases like FCS and may, in the future, address residual risk related to TGs.

##### 3) Plozasiran

The SHASTA-two phase two b randomized clinical trial evaluated plozasiran, an ApoC-III-targeted siRNA, in 229 adults with severe hypertriglyceridemia defined as fasting TG levels between 500 and 4,000 mg/dL [43]. Participants were randomized in a 3:1 ratio to receive plozasiran at doses of 10, 25, or 50 mg subcutaneously on day 1 and week 12, or placebo [43]. A total of 226 patients were included in the analysis, with a mean age of 55 years, and follow-up continued for 48 weeks. The primary outcome was the percentage change in TG levels at 24 weeks [43]. Plozasiran produced substantial and statistically significant reductions in TG concentrations, with a least-squares mean decrease of 57% at the highest dose (95% CI, −71.9% to −42.1%; P < 0.001) [43]. Reductions in ApoC-III of 77% were also observed (95% CI, −89.1% to −65.8%; P < 0.001), and over 90% of treated patients achieved TG levels below 500 mg/dL. LDL-C rose in a dose-dependent manner, reaching significance at the highest dose, while non-HDL-C decreased and ApoB remained stable. Adverse event rates were comparable between groups, with reported events generally mild to moderate and not considered treatment-related, and no deaths were recorded. The investigators concluded that plozasiran achieved robust and durable TG lowering with a favorable safety profile, though longer outcome trials will be necessary to determine its impact on clinical endpoints such as pancreatitis and ASCVD [43].

### CETP inhibitors

CETP inhibitors have had a long and checkered history in lipid management [44]. CETP transfers cholesterol from HDL to LDL/VLDL particles; inhibiting CETP raises HDL-C and can lower LDL-C [44]. Earlier CETP inhibitors (torcetrapib, dalcetrapib, evacetrapib) famously failed in outcomes trials due to off-target toxicity (torcetrapib raised blood pressure and mortality) or lack of efficacy in reducing cardiovascular events despite raising HDL [44].

#### Anacetrapib

Zhou et al performed a systematic review and meta-analysis of 10 RCTs involving 34,781 dyslipidemic individuals to evaluate the efficacy and safety of anacetrapib [45]. The drug induced significant alterations in lipid profiles: it elevated HDL-C (WMD 53.07 mg/dL, 95% CI, 46.79–59.36, P < 0.00001) and ApoA-I (WMD 53.44 mg/dL, 95% CI, 45.72–61.16, P < 0.00001), while markedly reducing LDL-C (WMD −32.99 mg/dL, 95% CI, −37.13 to −28.86, P < 0.00001), non-HDL-C (WMD −39.19 mg/dL, 95% CI, −52.22 to −26.16, P < 0.00001), TGs (WMD −9.97 mg/dL, 95% CI, −10.54 to −9.41, P < 0.00001), ApoB (WMD −22.55 mg/dL, 95% CI, −28.56 to −16.54, P < 0.00001), and Lp(a) (WMD −13.35 mg/dL, 95% CI, −18.31 to −8.39, P < 0.00001) [45]. Notably, adverse effects such as hepatotoxicity (OR 0.90, 95% CI, 0.75–1.07, P = 0.23), musculoskeletal injury (OR 1.01, 95% CI, 0.88–1.15, P = 0.90), drug-related adverse events (OR 1.00, 95% CI, 0.96–1.05, P = 0.99), and treatment withdrawals (OR 1.01, 95% CI, 0.95–1.08, P = 0.78) exhibited no significant difference compared with placebo [45]. The study indicated that anacetrapib significantly improved lipid profile by elevating HDL-C and reducing LDL-C and TGs, while exhibiting a safety profile similar to placebo, highlighting its potential as an effective lipid-modifying medication [45].

#### Obicetrapib

Obicetrapib is a next-generation, oral CETP inhibitor highlighted by Kastelein et al in a narrative review synthesizing data from phase two and three randomized trials in adults with dyslipidemia, most of whom were receiving high-intensity statin therapy and were at elevated risk of ASCVD [46]. In the phase two ROSE program, when added to background statins, obicetrapib produced substantial reductions in LDL-C of approximately 45% to 51% compared with baseline, with all effects highly significant (P < 0.001), accompanied by notable increases in HDL-C [46]. In the phase three BROOKLYN trial, which enrolled patients with heterozygous FH on maximally tolerated lipid-lowering therapy, obicetrapib achieved a mean LDL-C reduction of 36.3% compared with placebo at day 84 (P < 0.0001), and this effect was sustained at 1 year with a 41.5% reduction relative to placebo (P < 0.0001) [46]. Safety outcomes across studies were generally comparable to placebo, with adverse events mostly mild and no signal of the off-target toxicity that undermined earlier CETP inhibitors such as torcetrapib [46]. The current body of evidence indicates that obicetrapib produces robust improvements in atherogenic lipid parameters, including LDL-C, non-HDL-C, apoB, and Lp(a), while maintaining a favorable tolerability profile. These findings support CETP inhibition with obicetrapib as a promising adjunct to statins, pending confirmation of cardiovascular outcome benefits in ongoing phase three trials [46].

### Omega-3 fatty acid derivatives

#### Icosapent ethyl (IPE)

IPE, a highly purified ethyl ester of EPA, given at a dose of 4 g/day, demonstrated a landmark advance in cardiovascular prevention through the REDUCE-IT trial [47]. In statin-treated patients with elevated TGs (150–500 mg/dL) and either established CVD or diabetes with additional risk factors, IPE significantly reduced ischemic events [47]. The trial reported a 25% RR reduction in the primary composite outcome of MACEs and a 20% reduction in cardiovascular death compared with placebo [47]. These findings corresponded to an absolute risk reduction of approximately 4.8% over 5 years, leading the trial chair to describe the results as perhaps the biggest development in cardiovascular prevention since statins [47].

Although IPE lowered TGs by 18%, its benefits are thought to extend beyond TG reduction, potentially involving plaque stabilization, anti-inflammatory effects, membrane stabilization, and antithrombotic properties [47]. Importantly, prior studies of mixed omega-3 formulations containing both EPA and DHA (the STRENGTH trial) failed to demonstrate cardiovascular benefit, underscoring the significance of IPE’s unique, high-dose pure EPA formulation [47].

Following REDUCE-IT, professional guidelines endorsed IPE as adjunctive therapy in high-risk patients with controlled LDL-C but residual hypertriglyceridemia, particularly for secondary prevention [47]. Clinically, IPE is generally well tolerated; however, it has been associated with a slight increase in atrial fibrillation risk and a modest, non-significant increase in bleeding events [47]. Importantly, physicians should counsel patients that over-the-counter fish oil supplements are not interchangeable with prescription IPE, as supplements lack the purity, dosing, and demonstrated cardiovascular benefit of the REDUCE-IT regimen [47].

The success of IPE has reinvigorated interest in EPA-based therapies and structural analogues, as well as potential combination strategies with other lipid-lowering agents [47]. Overall, IPE has established omega-3 fatty acid therapy as an evidence-based option for residual cardiovascular risk reduction in the statin era, particularly among patients with persistent hypertriglyceridemia [47]. Summary of possible adverse events associated with pharmacotherapy listed above (Table 1) and a consolidated summary of studies on lipid pharmacotherapies is displayed in Table 2 [26–38, 40–43, 45–49].

**Table 1:** Summary of Possible Adverse Events Associated With Pharmacotherapy

Pharmacotherapy	Adverse effects
Bempedoic acid	Gout, cholelithiasis, small increases in serum creatinine, uric acid, and hepatic enzymes
Inclisiran	Most frequent events are injection-site reaction
Evinacumab	Diarrhea, dyspepsia, toothache, dizziness, nasopharyngitis, influenza-like illness, urinary tract infection, increased aspartate aminotransferase (AST), myalgia, suicide attempt (rare)
Zodasiran	Transient elevation in glycated hemoglobin (HbA1c) with pre-existing diabetes
Volanesorsen	Injection-site reactions, thrombocytopenia
Olezarsen	Headache, injection-site reactions, upper respiratory infections, liver enzyme elevation, Thrombocytopenia, decrease in estimated glomerular filtration rate
Plozasiran	Upper respiratory tract infection, headache, abdominal pain, diarrhea, urinary tract infection
Anacetrapib	Mild increase in blood pressure, diarrhea, constipation, dyspepsia, myalgias
Icosapent ethyl	Diarrhea, increased atrial fibrillation/flutter, bleeding risk, peripheral edema

**Table 2:** A Consolidated Summary of Studies on Lipid Pharmacotherapies

Author	Year	Study type	Sample size	Intervention	Duration	Key clinical outcome
Lincoff et al [26]	2024	Double blind, placebo-controlled trial	13,970	Bempedoic acid vs. placebo	40.6 months	Bempedoic acid reduced major adverse cardiovascular events by 15% compared to placebo.
Nicholls et al [27]	2024	Randomized, double-blind, placebo-controlled, clinical trial	13,970	Bempedoic acid vs. placebo	3.4 years	Bempedoic acid was associated with a 20% reduction in the total number of major cardiovascular events compared to placebo in high-risk, statin-intolerant patients.
Bays et al [28]	2025	Subset analysis of randomized, double-blind, placebo-controlled, clinical trial	13,970	Bempedoic acid vs. placebo	40.7 months	Bempedoic acid resulted in a placebo-corrected reduction in LDL-C of 22.5% and hs-CRP of 23.2%, and a 23% reduction in the composite MACE-4 endpoint compared with placebo in people with obesity.
Duell et al [29]	2024	Pooled patient-level data analysis of two phase 3 randomized clinical trials	217	Bempedoic acid vs. placebo	52 weeks	In patients with HeFH, adding bempedoic acid to maximally tolerated statin therapy resulted in a significant LDL-C reduction of −21.2% at week 12 versus placebo.
Taub et al [30]	2025	Randomized, double-blind, phase 3 trial	350	Inclisiran vs. ezetimibe vs. placebo	6 months	Inclisiran monotherapy reduced LDL-C by 46.5% at day 150 vs. placebo (1.4% increase) and vs. ezetimibe (−11.2%).
Basit et al [31]	2025	Meta-analysis of randomized controlled trials	5,016	Inclisiran vs. placebo	Trials included up to July 2024	Inclisiran significantly reduced LDL-C by a mean difference of −50.42% compared with control, without increasing serious adverse event risk.
Saad Cleto et al [32]	2025	Systematic review and meta-analysis	30,718	Inclisiran vs. alirocumab	24 weeks	Alirocumab demonstrated greater efficacy than inclisiran in enhancing lipid parameters.
Rai et al [33]	2024	Systematic review and meta-analysis		Inclisiran		The meta-analysis found significantly greater reductions in lipid-parameters in HeFH compared to HoFH.
Rangwala et al [34]	2024	Systematic review and meta-analysis	270	Evinacumab vs. placebo		High dose evinacumab significantly reduce lipid parameters with no excess adverse events.
Rosenson et al [35]	2020	Randomized, double-blind, placebo-controlled Phase 2 clinical trial	272	Evinacumab vs. placebo	16 weeks	Evinacumab reduced LDL-cholesterol by more than 50% at the highest dose compared to placebo.
Rosenson et al [36]	2024	Phase 2b, randomized, double-blind, placebo-controlled trial	204	Zodasiran vs. placebo	36 weeks	Zodasiran resulted in dose-dependent triglyceride reductions.
Kim et al [37]	2024	Systematic review and meta-analysis	53,231	Fibrate vs. placebo		Fibrate therapy was linked to a lower incidence of major cardiovascular events, with its beneficial impact primarily attributed to reductions in LDL-cholesterol rather than triglyceride levels.
Jakob et al [38]	2016	Systematic review and meta-analysis	16,135	Fibrate vs. placebo	4.8 years	Fibrate therapy lowered the combined risk of cardiovascular-disease death, non-fatal myocardial infarction, or non-fatal stroke in primary prevention.
Sahebkar et al [40]	2017	Systematic review and meta-analysis	1,388	Fibrate vs. statin		Fibrate therapy led to a markedly greater decrease in plasma lipoprotein(a) levels than statin treatment.
Gouni-Berthold et al [41]	2021	Randomized, double-blind, placebo-controlled phase 3 clinical trial	114	Volanesorsen vs. placebo	26 weeks	Volanesorsen resulted in a 71.2% decrease in triglyceride levels, compared to only a 0.9% change observed in the placebo group.
Bergmark et al [42]	2024	Randomized, double-blind, placebo-controlled, phase 2b trial	154	Olezarsen vs. placebo	6 months with follow-up up to 12 months	Olezarsen at doses of 50 and 80 mg produced triglyceride reductions of approximately 49.3% and 53.1%, respectively, when compared with the placebo group.
Gaudet et al [43]	2024	Randomized clinical trial	226	Plozasiran vs. placebo	48 weeks	Plozasiran produced robust triglyceride lowering, with a least-squares mean reduction of 57% at the highest dose; ApoC-III decreased by 77%, and more than 90% of treated participants achieved triglyceride levels below 500 mg/dL.
Zhou et al [45]	2018	Systematic review and meta-analysis of randomized controlled trials	34,781	Anacetrapib vs. placebo		Anacetrapib significantly improved lipid parameters, increasing HDL-C and reducing LDL-C, non-HDL-C, triglycerides, ApoB, and Lp(a), without a significant increase in adverse events compared with placebo.
Kastelein et al [46]	2024	Narrative review of phase 2 and phase 3 randomized trials	_	Obicetrapib vs. placebo/statin	_	Obicetrapib produced substantial LDL-C reductions of approximately 45–51% in phase 2 studies and 36.3% at day 84 in the phase 3 BROOKLYN trial, with sustained benefit and favorable tolerability.
Sayah et al [47]	2024	Randomized clinical trial	_	Icosapent ethyl vs. placebo	5 years	Icosapent ethyl reduced the primary composite cardiovascular endpoint by 25% and cardiovascular death by 20% in statin-treated patients with elevated triglycerides and high cardiovascular risk.
Hafiane et al [48]	2025	Phase 3 trial	17	Lomitapide vs. baseline		Lomitapide significantly reduced LDL-C and ApoB in patients with HoFH, with larger reductions at higher doses and associated changes in HDL function.
Karwatowska-Prokopczuk et al [49]	2023	Randomized, double-blind, placebo-controlled phase 1 study	29	Pelacarsen vs. placebo	204 days	Pelacarsen produced marked dose-dependent reductions in lipoprotein(a), reaching up to approximately 84% with repeated dosing, with acceptable short-term safety.

HeFH: heterozygous familial hypercholesterolemia; HoFH: homozygous familial hypercholesterolemia; hs-CRP: high-sensitivity C-reactive protein; LDL: low-density lipoprotein; MACEs: major adverse cardiovascular events.

## Nutraceuticals and Dietary Interventions

Lifestyle modification remains the foundation of lipid management, and there is renewed interest in nutraceuticals and specific diets as adjuncts or alternatives to pharmaceuticals.

### Red yeast rice (RYR)

RYR is produced by fermenting rice with *Monascus purpureus*, generating bioactive monacolins that act as weak inhibitors of HMG-CoA reductase, thereby reducing endogenous cholesterol synthesis [50]. Clinical studies have demonstrated that RYR, administered at doses ranging from 1.2 to 4.8 g/day, lowers LDL-C, total cholesterol, TGs, and HDL-C [50]. In a large secondary prevention trial of post-MI patients, RYR therapy resulted in an approximate 20% reduction in LDL-C, a 4.7% absolute risk reduction in major coronary events, and a 30% reduction in cardiovascular mortality over a mean follow-up of 4.5 years [50].

Despite these benefits, safety concerns remain. The principal risks are related to monacolin K-mediated inhibition of CYP450 enzymes and potential impurities formed during fermentation, particularly citrinin, a nephrotoxin [50]. Notably, an RYR supplement produced by Kobayashi Pharmaceutical Company in Japan was associated with 114 hospitalizations and five deaths, attributed to contamination with puberulic acid, raising concern over the quality control of commercial preparations [51].

### Plant sterols and stanols

These compounds, found in fortified foods and supplements (e.g., margarines or pills), reduce intestinal cholesterol absorption [52]. A typical dose (2 g/day) can lower LDL-C by 5–15%. They are considered safe and have been incorporated into dietary guidelines for cholesterol management. While they do not have outcome trials of their own, their LDL-lowering effects contribute to overall risk reduction [52].

### Viscous (soluble) fiber

The study based on a comprehensive evaluation of RCT. Adults with or without hypercholesterolemia who received psyllium (median dose 10.2 g/day) for at least 3 weeks were the subjects of 28 trials (n = 1,924) [53]. Reductions in LDL-C (MD −0.33 mmol/L; 95% CI, −0.38 to −0.27; P < 0.00001), non-HDL-C (MD −0.39 mmol/L; 95% CI, −0.50 to −0.27; P < 0.00001), and apoB (MD −0.05 g/L; 95% CI, −0.08 to −0.03; P < 0.0001) were significantly lower than the control group [53]. In summary, the evidence quality was evaluated as good for apoB and intermediate for LDL-C and non-HDL-C, indicating that psyllium fiber successfully improves both conventional and alternative atherogenic lipid markers, lending credence to its usage in reducing the CVD risk associated with atherosclerosis [53]. Psyllium fiber is sometimes recommended as an adjunct for patients who need only mild LDL lowering, or as part of an overall heart-healthy diet [53].

### Bergamot and berberine

Lamiquiz-Moneo and colleagues conducted a systematic review of human studies evaluating the effects of bergamot (*Citrus bergamia*) on lipid outcomes [54]. Of the 442 records screened, 12 interventional and observational studies were included [54]. Approximately 75% of these trials reported statistically significant reductions in atherogenic lipids, with decreases in total cholesterol ranging from 12.3% to 31.3%, LDL-C from 7.6% to 40.8%, and TGs from 11.5% to 39.5%, while eight trials also documented increases in HDL-C [54].

The author performed a systematic review and meta-analysis of 16 randomized clinical trials including 2,147 participants to assess the efficacy and safety of berberine in the management of dyslipidemia [55]. The pooled results demonstrated significant improvements in lipid parameters, with total cholesterol reduced by −0.47 mmol/L (95% CI, −0.64 to −0.31, P < 0.00001), LDL-C reduced by −0.38 mmol/L (95% CI, −0.53 to −0.22, P < 0.00001), and TGs reduced by −0.28 mmol/L (95% CI, −0.46 to −0.10, P = 0.002) [55]. When administered as monotherapy, berberine also increased HDL-C by 0.08 mmol/L (95% CI, 0.03 to 0.12, P = 0.001) [55]. With respect to safety, berberine was generally well tolerated and did not show a significant increase in overall adverse events compared with control groups (RR 0.64, P = 0.22), and no severe adverse events were reported. The most frequently observed adverse reactions were mild gastrointestinal complaints, such as constipation, diarrhea, and abdominal discomfort, though these did not occur more often than in the placebo arms [55]. Overall, the findings suggest that berberine can improve lipid profiles while maintaining an acceptable short-term safety profile; however, the strength of the evidence is limited by study heterogeneity and methodological weaknesses [55].

### Turmeric/curcumin

The root of *Curcuma longa* is the source of the spice turmeric. Curcumin, the principal bioactive compound, has been identified as the most potent constituent because it enhances the activity of cholesterol 7α-hydroxylase, the key enzyme that converts cholesterol into bile acids, which are subsequently excreted [56]. Evidence regarding the effect of turmeric on lipid profiles remains inconclusive. Findings from several meta-analyses suggest only modest benefits, with reported average reductions of approximately 4 mg/dL (0.103 mmol/L) in total cholesterol, 7 mg/dL (0.079 mmol/L) in TGs, and less than 5 mg/dL (0.129 mmol/L) in LDL-C. At present, limited data are available on whether turmeric supplementation influences CVD risk [56]. Reported adverse effects are generally mild and include gastrointestinal symptoms such as bloating, flatulence, and diarrhea [56].

### Supplements vs. statins

Laffin et al conducted a randomized, double blind trial in 190 adults without ASCVD to compare low-dose rosuvastatin with placebo and six dietary supplements (fish oil, cinnamon, garlic, turmeric, plant sterols, and RYR) [57]. Over 28 days, rosuvastatin 5 mg daily reduced LDL-C by 37.9% (P < 0.001), along with significant decreases in total cholesterol and TGs, while none of the supplements or placebo produced meaningful lipid or inflammatory biomarker changes. The trial confirmed that statins remain far more effective than commonly used lipid-lowering supplements, with a comparable short-term safety profile across groups [57].

### Dietary patterns

For lipid management, the Portfolio Diet and the Mediterranean (Med) diet offer different strengths. In a randomized, metabolically controlled trial, the Portfolio Diet combining plant sterols, viscous fiber, soy protein, and almonds achieved 28–30% LDL-C reductions within 4 weeks, a magnitude comparable to low-dose statin therapy and significant vs. control (P < 0.001) [58]. In contrast, the Med diet’s primary evidence comes from PREDIMED, where diets supplemented with extra virgin olive oil or nuts reduced major cardiovascular events versus a reduced-fat diet; accompanying lipid changes were modest (generally small but significant decreases in LDL-C and TGs and a small HDL-C increase), suggesting that outcome benefits extend beyond lipids alone [59]. It is important to note that nutraceuticals and diets usually produce moderate lipid changes and should not be viewed as replacements for evidence-based therapies in high-risk patients. However, they can be valuable for low-risk individuals with mild dyslipidemia, or as adjuncts to medication in those seeking maximal lifestyle optimization.

## Gene-Editing Therapies (CRISPR-Based LDL Modulation)

Perhaps the most groundbreaking frontier in lipid management is *in vivo* gene editing to permanently alter lipid-related genes. The target that has moved fastest into the clinic is PCSK9, the same protein targeted by monoclonal antibodies and inclisiran [60]. Using CRISPR-based technology, researchers have developed a way to permanently inactivate the *PCSK9* gene in liver cells, aiming for a one-time treatment that provides lifelong LDL-C reduction. In 2022, a biotech company reported the first human trial of a base-editing therapy, VERVE 101, in patients with heterozygous FH [60]. This therapy uses a modified CRISPR system to introduce a single base change in the *PCSK9* gene, rendering it inactive. Preliminary results (presented in 2023) are very promising: a single infusion of VERVE 101 led to substantial and lasting reductions in LDL-C and PCSK9 levels [60]. Among the first patients treated, those receiving higher doses saw LDL-C reductions of approximately 40–55% from baseline, along with a 47–84% drop in circulating PCSK9 protein levels. These findings provide the first proof-of-concept that gene editing can safely and effectively lower cholesterol in humans [60]. The implications are enormous; it hints at a future where an inherited predisposition to high LDL could be corrected with a one-time therapy, obviating the need for lifelong medications.

## Combination Therapies and Patient Selection

The therapeutic strategy in lipid management involves combining agents with complementary mechanisms to achieve additive reductions in LDL-C. A high-intensity statin alone generally lowers LDL-C by about 50% [61]. When ezetimibe is added, an additional reduction of roughly 15% can be obtained, while PCSK9 inhibitors as monotherapy typically achieve decreases of approximately 60% [61]. When used in combination, statins, ezetimibe, and PCSK9 inhibitors can collectively reduce LDL-C by up to 85% from baseline, allowing many patients particularly those at very high or extreme cardiovascular risk to achieve targets below 55 mg/dL [61]. The central principle is to individualize therapy according to both risk profile and treatment tolerance, selecting the combination that delivers optimal lipid lowering without undue adverse effects [61].

### Patient selection for these novel therapies

#### Statin-intolerant patients

These patients cannot tolerate adequate statin doses due to muscle pain or other adverse effects. For them, non-statin options become frontline [62]. Bempedoic acid (oral) and PCSK9 inhibitors, or inclisiran (injectable), are key alternatives. For example, a statin-intolerant patient with ASCVD could be managed with bempedoic acid plus ezetimibe and, if needed, inclisiran or a PCSK9 mAb to reach the goal [62]. Statin intolerance should be evaluated when patients develop persistent muscle-related symptoms, unexplained elevations in creatine kinase (CK), or intolerance to at least two different statins, including one at the lowest approved dose. Clinical assessment begins with careful review of symptom timing in relation to statin initiation, dose escalation, or drug interactions. Laboratory evaluation typically includes serum CK to assess myopathy, liver transaminases (ALT/AST) to evaluate hepatic effects, and thyroid-stimulating hormone and vitamin D levels to exclude secondary contributors to myalgia. When symptoms occur, temporary statin discontinuation followed by rechallenge with an alternative statin, lower dose, or intermittent dosing strategy is recommended to confirm true intolerance before considering non-statin lipid-lowering therapies [15, 61, 62].

#### Very high-risk ASCVD patients

Patients with recent events or polyvascular disease often require maximal LDL-lowering. Here, adding a PCSK9 inhibitor on top of statin/ezetimibe is recommended if LDL is above goal (e.g., > 70 mg/dL); this is supported by outcomes trials such as FOURIER and ODYSSEY [63]. Inclisiran can be an alternative to PCSK9 mAbs in this setting, given its similar LDL-lowering effect. If the patient still has LDL > 55 mg/dL on those, one might even consider adding bempedoic acid or other agents, although data on triple or quadruple therapy combinations are limited [63]. The guiding principle is that lower LDL levels are better in high-risk patients, so aggressive combinations are justified [63].

#### FH

FH is an inherited disorder characterized by markedly elevated LDL-C levels and a substantially increased risk of premature ASCVD. Early identification of affected individuals is essential because timely lipid-lowering therapy can significantly reduce cardiovascular morbidity and mortality [21]. Heterozygous FH affects approximately 1 in 200–250 individuals worldwide, whereas homozygous FH is considerably rarer, with an estimated prevalence of approximately 1 in 160,000–300,000 individuals [21, 24]. To standardize clinical diagnosis, validated algorithms such as the Simon Broome criteria and the Dutch Lipid Clinic Network (DLCN) criteria are widely used in clinical practice and research [21, 64]. The clinical diagnosis of FH commonly relies on established diagnostic frameworks such as the Simon Broome criteria and the DLCN criteria, both of which integrate biochemical findings with clinical and familial characteristics. The Simon Broome criteria, developed in the United Kingdom, classify individuals as having definite or possible FH based on a combination of lipid measurements, physical findings, genetic testing, and family history. Markedly elevated total cholesterol or LDL-C levels serve as the initial screening indicator. A definite diagnosis is established when elevated cholesterol levels are accompanied by tendon xanthomas in the patient or a first-degree relative, or when a pathogenic mutation is identified in genes involved in LDL metabolism, including LDLR, APOB, or PCSK9. In the absence of these findings, individuals with significant hypercholesterolemia and a family history of premature coronary artery disease or hypercholesterolemia may be classified as having possible FH [21]. Owing to its straightforward structure and clinical practicality, the Simon Broome system is widely applied in routine clinical assessment.

In contrast, the DLCN criteria use a structured point-based scoring system to estimate the likelihood of FH. Points are assigned across several domains, including family history of premature CVD or hypercholesterolemia, personal history of premature ASCVD, characteristic physical signs such as tendon xanthomas or early corneal arcus, measured LDL-C concentration, and confirmation of a pathogenic genetic mutation. The cumulative score stratifies individuals into diagnostic categories of definite FH (≥ 8 points), probable FH (6–8 points), possible FH (3−5 points), or unlikely FH (< 3 points). By providing graded diagnostic probability, the DLCN model facilitates standardized assessment in both clinical practice and epidemiological studies, particularly within specialized lipid clinics and screening programs for FH [21].

Heterozygous FH patients often require multiple drugs. A common regimen would be high-intensity statin plus ezetimibe plus PCSK9 inhibitor, which often achieves > 60% LDL reduction [64]. If more is needed, inclisiran could substitute or rotate with PCSK9 therapy. In homozygous FH, standard therapy is less effective; these patients often end up on combinations including statin, ezetimibe, lomitapide (a specialized microsomal transfer protein inhibitor), and now evinacumab (ANGPTL3 antibody), plus LDL apheresis in some cases [64]. Evinacumab is a game changer for homozygous FH, often added to whatever baseline therapy they can tolerate, given its unique mechanism [64].

#### Lomitapide

Hafiane et al report an open-label analysis of stored samples from 17 patients with homozygous FH enrolled in the lomitapide phase-three program to assess HDL metrics and cholesterol efflux alongside standard lipids [48]. Compared with baseline, LDL-C and apoB fell significantly (P < 0.01), with larger reductions at higher lomitapide doses; Lp(a) declined only at the higher dose (about −27%, versus –55% for LDL-C and apoB). HDL-C rose modestly (4.2%), while apoA-I fell slightly (−3%) [48]. Functionally, total cholesterol-efflux capacity and ABCA1-mediated efflux decreased, whereas SR-BI-mediated cholesterol uptake increased in a dose-dependent manner (21.4% at lower and 64.3% at higher doses), with ABCG1 showing no consistent change [48]. The authors conclude that lomitapide may promote HDL particle lipidation through an SR-BI dependent process, shifting reverse cholesterol transport dynamics and attenuating total efflux, even as atherogenic lipoproteins are substantially lowered [48].

#### Elevated TG and mixed dyslipidemia

For patients with TG-rich dyslipidemia (e.g., diabetic dyslipidemia with high TG, low HDL), IPE has a clear role if they meet criteria (TG ≥ 150 mg/dL, established CVD or high risk). Fibrates or high-dose omega-3 may be considered for very high TG (> 500) to prevent pancreatitis, but fibrates have not shown CV benefit when added to statins. New ApoC-III or ANGPTL3 inhibitors could be considered in specialized cases of refractory hypertriglyceridemia or in clinical trials [34, 37, 42, 63].

#### Lp(a) elevation

In patients with elevated Lp(a), contemporary evidence supports a pragmatic approach that leverages therapies with modest Lp(a)-lowering while awaiting targeted agents. In FOURIER, addition of evolocumab to statin therapy led to a 20–25% reduction in Lp(a), and greater on-treatment Lp(a) lowering correlated with fewer cardiovascular events, supporting use of PCSK9 inhibitors partly for this ancillary benefit in very high-risk patients [10, 17]. Authoritative reviews also note that niacin can modestly reduce Lp(a), though it has not demonstrated cardiovascular outcome benefit and is limited by tolerability; meanwhile, antisense/siRNA therapies targeting Lp(a) are advancing and may soon offer substantial, mechanism-specific reductions [10, 17].

#### Pelacarsen

Pelacarsen is a liver-targeted antisense oligonucleotide conjugated to N-acetylgalactosamine that binds to LPA messenger RNA in hepatocytes and induces RNase H1-mediated degradation, which reduces apolipoprotein(a) synthesis and thereby lowers plasma concentrations of Lp(a) [49]. In this randomized double blind placebo-controlled phase-one study, 29 healthy Japanese adults received either a single dose of 20, 40, or 80 mg or multiple monthly doses of 80 mg, with safety, pharmacokinetics, and exploratory efficacy assessed over follow-up to day 204 [49]. The abstract does not report a mean age. In the single dose groups, the placebo-corrected least squares mean change in Lp(a) at day 30 was −55.4% with P = 0.0008 for 20 mg, −58.9% with P = 0.0003 for 40 mg, and 73.7% with P < 0.0001 for 80 mg [49]. In the multiple-dose cohort, reductions were −84.0% at day 29 (P = 0.0003) and reached 106.2% at day 85 (P < 0.0001), with sustained but gradually attenuated lowering through day 204. Pharmacokinetics showed rapid absorption with peak concentrations at about 4 h, and biexponential elimination [49]. Safety was acceptable, with no serious adverse events and no clinically meaningful laboratory abnormalities. The investigators concluded that pelacarsen was well tolerated and produced potent reductions in Lp(a) in this Japanese population, supporting further evaluation of the 80 mg monthly regimen in outcomes studies [49]. Table 3 summarizes the target, primary mechanism, and lipid action pharmacotherapies discussed in this review.

**Table 3:** Medications Summary

Drug	Target	Primary mechanism	LDL-C	TG	HDL-C
Statin	HMG-CoA reductase	Blocks cholesterol synthesis	↓↓↓	↓	↑
PCSK9 inhibitor	PCSK9 protein	Increases LDL receptor recycling	↓↓↓	_	↑
Bempedoic acid	ACL enzyme	Blocks acetyl-CoA production	↓↓	_	_
Evinacumab	ANGPTL3 protein	Activates LPL and EL	↓↓	↓↓	↓
Zodasiran	ANGPTL3 mRNA	siRNA silences ANGPTL3	↓↓	↓↓	↓
ApoC-III inhibitor	ApoC-III protein	Enhances LPL and TG clearance	_	↓↓↓	↑
CETP inhibitor	CETP enzyme	Blocks CE transfer HDL→LDL	↓↓	↓	↑↑↑
Fibrates	PPARα receptor	Activates LPL, reduces TG synthesis	↓	↓↓↓	↑↑
Icosapent ethyl	Multiple (TG pathway)	Reduces TG, anti-inflammatory	_	↓↓	_
Lomitapide	MTP protein	Blocks VLDL assembly	↓↓↓	↓↓	↓
Pelacarsen	Apo(a) mRNA	ASO reduces Lp(a) production	_	_	_

↓↓↓: strong reduction; ↓↓: moderate reduction; ↓: mild reduction; ↑: increase; -: no significant effect. ACL: ATP-citrate lyase; ANGPTL3: angiopoietin-like protein 3; Apo(a): apolipoprotein(a); ApoC-III: apolipoprotein C-III; ASO: antisense oligonucleotide; CE: cholesterol ester; CETP: cholesteryl ester transfer protein; EL: endothelial lipase; HDL: high-density lipoprotein; HMG-CoA-3: hydroxy-3-methylglutaryl-CoA; LDL: low-density lipoprotein; LDLR: LDL receptor; Lp(a): lipoprotein(a); LPL: lipoprotein lipase; MTP: microsomal TG transfer protein; PCSK9: proprotein convertase subtilisin/kexin 9; PPARα: peroxisome proliferator-activated receptor α; siRNA: small interfering RNA; TG: triglyceride; VLDL: very low-density lipoprotein.

### Real-world implementation barriers and health-system challenges

Although statins are widely available as inexpensive generic medications, real-world implementation challenges also persist with these agents. Long-term adherence to statin therapy is frequently suboptimal; observational studies indicate that adherence rates may fall to 25–40% in some populations within the first year of therapy, particularly in primary prevention settings [65]. Several factors contribute to this pattern, including perceived or actual adverse effects such as myalgia, fear of medication-related complications, polypharmacy burden, and patient preference for lifestyle modification rather than long-term pharmacotherapy [66]. Financial barriers, although smaller than those associated with biologic therapies, can still affect adherence when copayments accumulate or when patients lack prescription coverage. In addition, inadequate patient education, inconsistent follow-up, and therapeutic inertia among clinicians may further reduce persistence with statin therapy over time [66].

For agents such as PCSK9 inhibitors and RNA-based therapies, high drug prices and insurance restrictions represent major obstacles. Early analyses reported annual list prices of approximately $14,000–$14,500 per year for PCSK9 inhibitors, prompting insurers to impose strict prior-authorization requirements and high patient copayments to control costs [67]. Consequently, access delays and prescription denials are common in routine practice; real-world studies report initial denial rates approaching 80%, with approval often requiring extensive documentation and repeated authorization processes [67]. Even when approved, out-of-pocket costs remain significant, particularly for patients enrolled in Medicare Part D, where monthly copayments can exceed $300 or annual copayments can exceed $5,000, contributing to prescription abandonment and reduced adherence [68]. Similar barriers affect emerging therapies such as inclisiran, ANGPTL3 inhibitors, and ApoC-III-targeting drugs, which remain costly and often require specialty pharmacy distribution, injectable administration, and insurance approval pathways that limit accessibility in routine practice [69].

## Strengths and Limitations

This review’s strengths are its integration of mechanistic biology with late phase clinical evidence to map therapies to specific patient phenotypes and its pragmatic framework for sequencing and combining agents in ways that mirror real-world workflows and contemporary guideline trends. At the same time, its narrative design limits formal comparative effectiveness and cost effectiveness conclusions across drug classes, and the heterogeneity of trial populations and endpoints constrains direct cross-trial comparisons of efficacy and safety. Together, these features yield a clinically useful synthesis while underscoring where structured head-to-head analyses and standardized outcomes are still needed to refine treatment selection.

## Future Directions

Future directions in lipid management emphasize head-to-head and pragmatic trials to compare combination strategies across statin-intolerant ASCVD, FH, and mixed dyslipidemia, along with cost-utility analyses to guide real-world access. Long-term safety and outcome data for RNA-based and ANGPTL3/ ApoC-III therapies are essential, while implementation research should focus on adherence through clinic-based injections, digital tools, and fixed-dose polypills. Exploration of synergistic regimens targeting LDL, TGs, and Lp(a), and careful advancement of gene editing approaches with safeguards for safety and equity, will shape the next era of precision lipid therapy. Machine-learning models integrated with electronic health records can assist clinicians in risk stratification, treatment adherence monitoring, and timely therapy intensification, thereby improving precision in dyslipidemia management and reducing preventable cardiovascular events.

## Conclusions

This review delineates a transition in lipid management from a statin-centric model to a diversified, mechanism-based armamentarium. In addition to PCSK9 monoclonal antibodies, bempedoic acid, inclisiran, ANGPTL-three and APOC-three directed agents, next-generation CETP inhibition, and high-dose EPA collectively expand the capacity to lower LDL-C, attenuate TG-rich lipoproteins, and address genetically mediated dyslipidemias. Among emerging agents, the most compelling outcome evidence comes from bempedoic acid in the CLEAR Outcomes trial and high-dose EPA in REDUCE-IT, while PCSK9 pathway therapies continue to demonstrate consistent LDL-C lowering with a favorable safety profile. Applied pragmatically, these therapies enable phenotype-guided care for statin-intolerant ASCVD, heterozygous and homozygous FH, mixed dyslipidemia, and severe hypertriglyceridemia, with realistic adherence advantages through less frequent dosing and simplified combinations.

Notwithstanding these advances, key evidence gaps remain. Cross-trial comparisons are limited by population heterogeneity and inconsistent endpoints; long-term safety and outcomes data are still maturing for RNA-based and ANGPTL3/ApoC-III targeted approaches; and access is uneven due to cost, prior authorization, and infrastructure needs for injectables. These constraints underscore the importance of rigorous head-to-head and pragmatic studies, standardized outcome reporting, and health-system strategies that close implementation gaps.

## Data Availability

The authors declare that data supporting the finding of this study are available within the article.

## Abbreviations

ANGPTL3: angiopoietin like protein 3
ApoC-III: apolipoprotein C-III
ATP: adenosine triphosphate
AST: aspartate amino transferase
ACL: ATP-citrate lyase
Apo(a): apolipoprotein(a)
ASO: antisense oligonucleotide
CE: cholesterol ester
CETP: cholesteryl ester transfer protein
CRISPR: clustered regularly interspaced short palindromic repeats
EPA: eicosapentaenoic acid
EL: endothelial lipase
HDL: high-density lipoprotein
HbA1c: hemoglobin-A1c
HMG-CoA: 3-hydroxy-3-methylglutaryl coenzyme A
hs-CRP: high-sensitivity C-reactive protein
LDL: low-density lipoprotein
LDLR: low-density lipoprotein receptor
mAb: monoclonal antibody
MTP: microsomal transfer protein
MACEs: major cardiovascular events
PCSK9: proprotein convertase subtilisin/kexin type 9
siRNA: small interfering ribonucleic acid
TGs: triglycerides
VLDL: very low-density lipoprotein
